# Mycetoma in a non-endemic area: a diagnostic challenge

**DOI:** 10.1186/s12907-017-0040-5

**Published:** 2017-02-02

**Authors:** Boubacar Efared, Layla Tahiri, Marou Soumana Boubacar, Gabrielle Atsam-Ebang, Nawal Hammas, El Fatemi Hinde, Laila Chbani

**Affiliations:** 1 Departement of Pathology, Hassan II Teaching Hospital, Fès, Morocco; 2 Departement of Parasitology, Hassan II Teaching Hospital, Fès, Morocco

**Keywords:** Actinomycetoma, Eumycetoma, Misdiagnosis, Pathology

## Abstract

**Background:**

Mycetoma is a chronic granulomatous infectious disease caused by filamentous bacteria or by fungi. The disease is endemic in certain tropical and subtropical areas of the world but can be found elsewhere posing sometimes a diagnostic challenge for clinicians.

**Case presentation:**

A 65-year- old man presented with a right foot swelling evolving for 25 years. During that time, several diagnosis and treatments have been made without any improvement. The disease spread to bones, and misdiagnosed as Kaposi’s sarcoma. Transtibial amputation has been performed, and the histopathological examination revealed finally the diagnosis of eumycotic mycetoma. The patient recovered well after surgery and orthopedic prosthesis was prescribed for him.

**Conclusion:**

Mycetoma in non endemic areas is usually misdiagnosed and mismanaged leading to unnecessary and inappropriate surgery. Health practitioners should be aware of that fact in order to provide an accurate management.

## Background

Madura foot or mycetoma is a chronic granulomatous disease of the subcutaneous tissue, that can progress to deeper structures like muscles or bones [1–3]. It is caused either by fungi (eumycetoma) or by aerobic filamentous bacteria (actinomycetoma) [1, 4]. It affects mostly lower extremities of the body, especially foot and leg but can affect any part of the body, such us head and neck, arms, the chest wall or the abdominal wall [1, 2, 5]. The disease often occurs in tropical and subtropical regions of the world, in the zone called “mycetoma belt”, extending between latitudes 15° south and 30° north [1, 3, 6]. Mexico, Senegal, India, Sudan, are the most affected countries [1–4]. Sudan seems to be the most endemic country where eumycetoma represents the main aetiologic type of the disease. This country hosts an important research center for mycetoma where large studies on the topic were performed [5]. But, in temperate climate, cases of mycetoma have been reported, mostly imported cases from immigrants [7–10]. In 2014, Buonfrate et al. had reported 42 cases of mycetoma acquired in Europe, through a literature review, suggesting that Europeans without travel history can be affected by the disease [11]. Typically, mycetoma is encountered in rural areas in poor people working in agricultural sector [1, 3, 5]. In 2013, the World Health Organisation (WHO) listed the disease among neglected tropical disease [12]. Several causative fungal or bacterial agents are responsible for the disease. The treatment is based on the type of causative agent, bacterial or fungal, and on the extent of the disease. Unfortunately, the diagnosis of the disease and the identification of the etiological agent is a very challenging issue, especially in non-endemic areas [13–16].

We report herein, a case of Madura foot evolving for more than 2 decades, that had escaped all diagnostic tools, misdiagnosed as cancer and leading finally to amputation. The final diagnosis has been achieved by the histopathological examination of the resected specimen.

## Case presentation

**Fig. 1:**
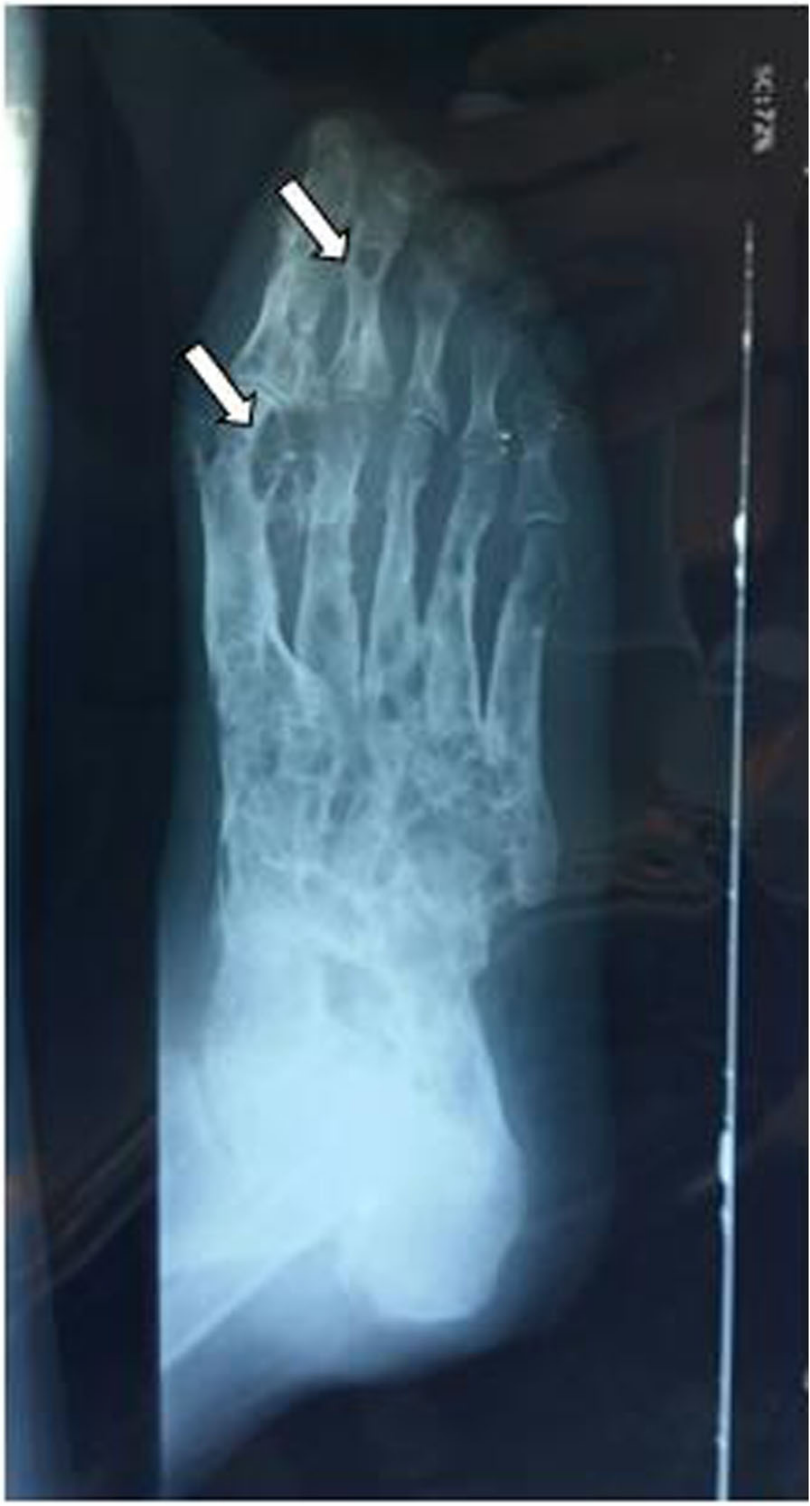
X rays of the foot showing extensive osteomyelitis with tarsal, metatarsal and phalange bones destruction (*arrows*)

A 65-year-old man was referred for evaluation of the right foot tumor diagnosed recently as Kaposi’s sarcoma. The patient was a shopkeeper living in the town of Fès and did not report any trip to an endemic area of mycetoma. He had a right foot chronic lesion for 25 years, with several repeated histological biopsies revealing, keloid scar, non specific inflammation, or Kaposi’s Sarcoma. The Physical examination showed chronic skin changes on the right foot and leg, with multiple scars and hard abscessed ulcerations on the plantar face of the foot. There were no grain discharge and the patient did not report such information. The culture of the abscess showed *Staphylococcus aureus* species. X-ray of the right foot was performed and showed extensive destruction of the tarse, metatarse and phalanges (Fig. 1). Other radiological evaluation did not found further lesions. The diagnosis of locally invasive Kaposi’s Sarcoma was suspected and a right trans-tibial amputation was performed.

### Histopathological findings

**Fig. 2:**
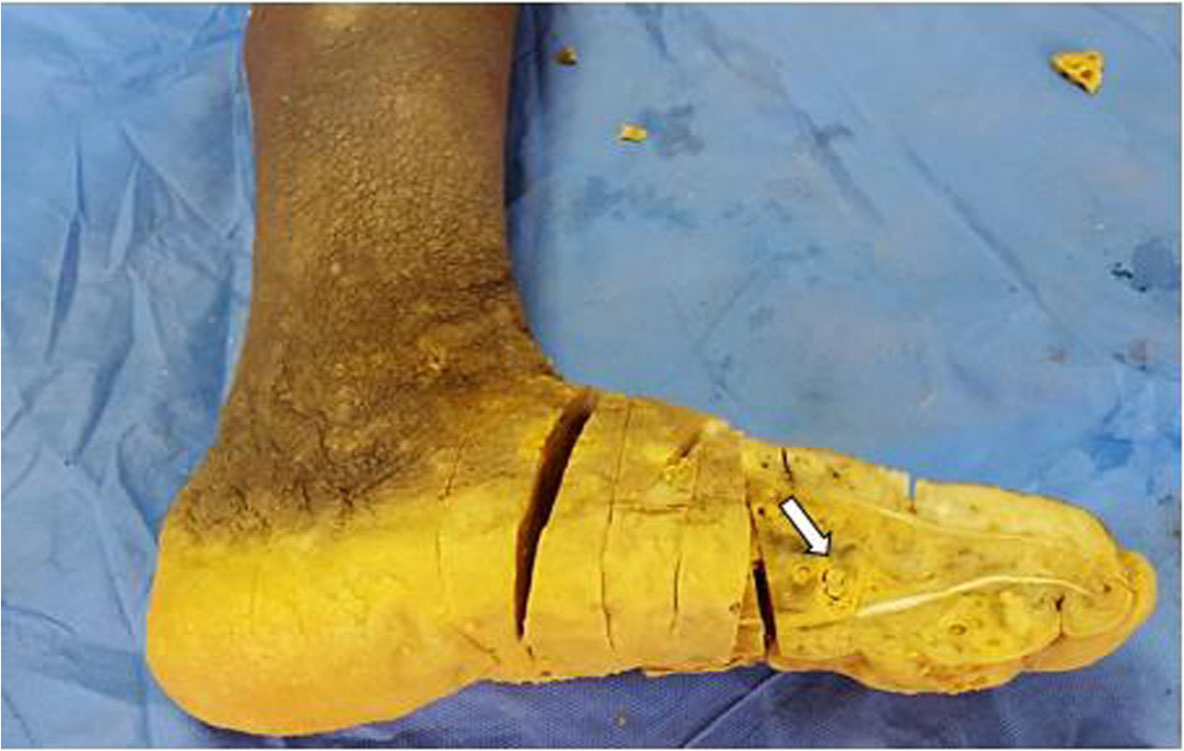
The resected specimen showing cavitis filled of yellowish materiel (*arrow*) corresponding to mycetoma grains

**Fig. 3:**
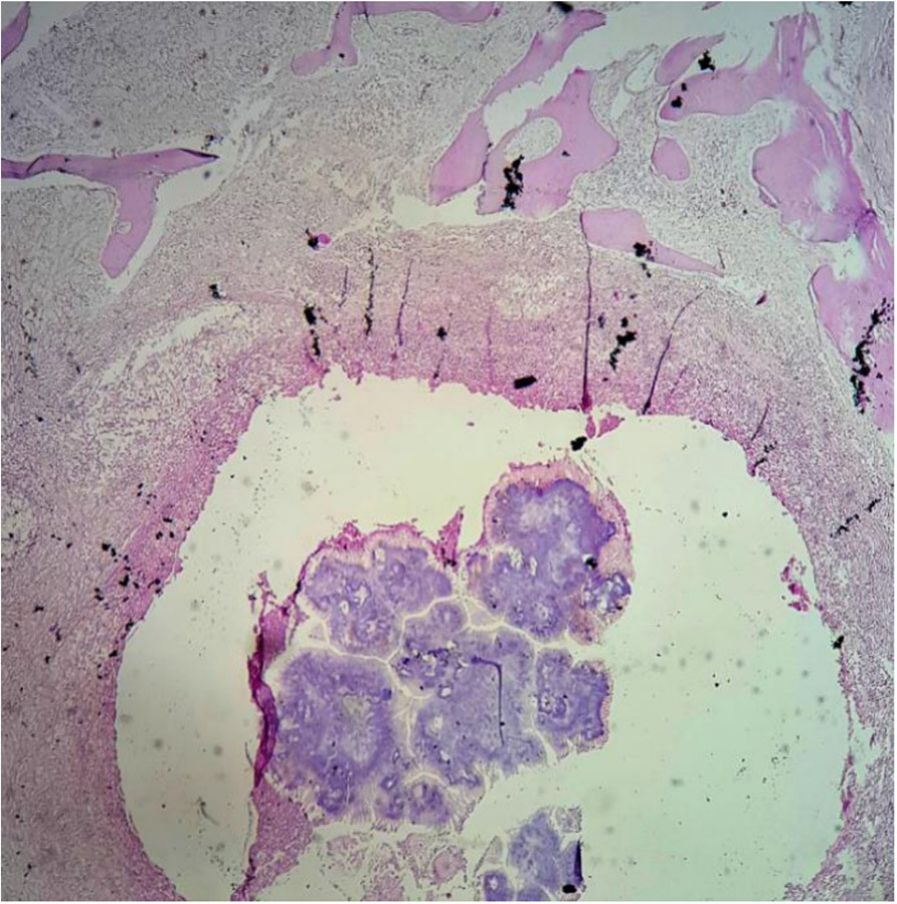
Histological aspects (HES stained section) with a fractured colony destroying the bone tissue

**Fig. 4:**
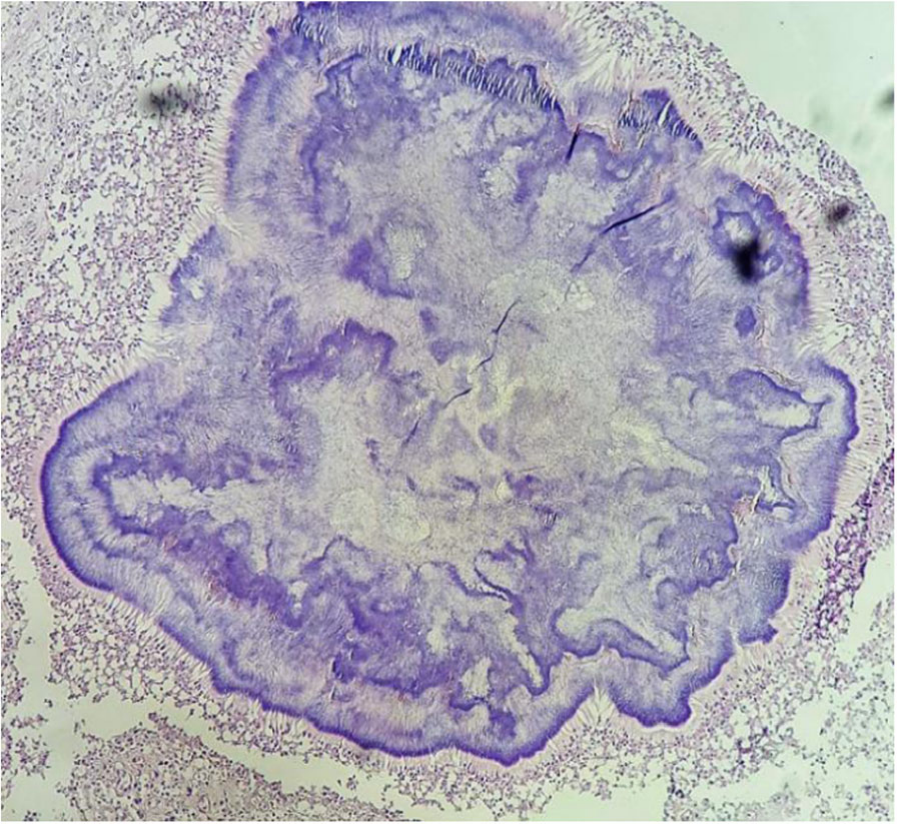
The histological image (HES stained section) showing a mycetoma colony with deeply basophilic outer layer and a pale center

**Fig. 5:**
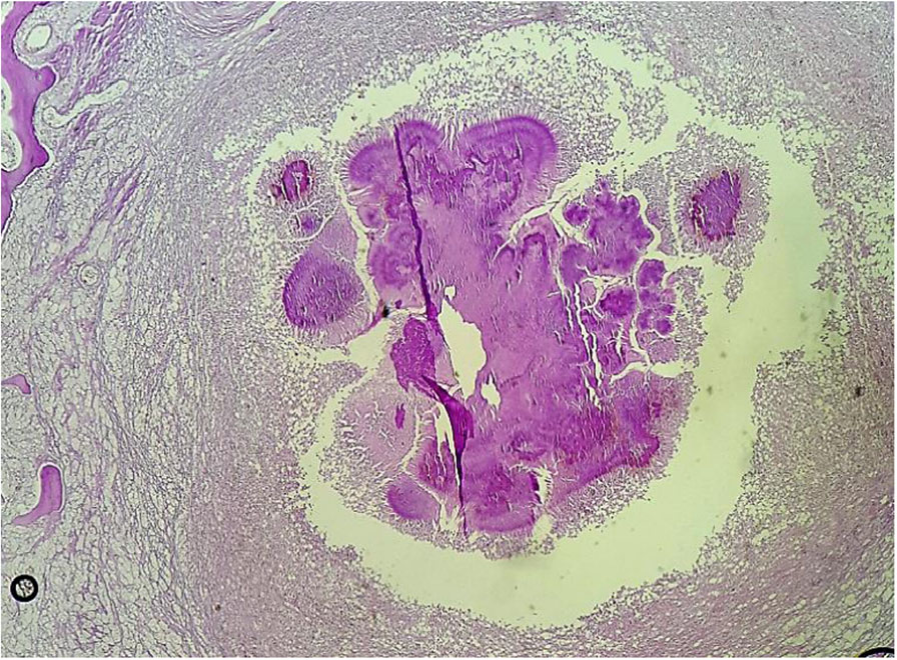
Histological image (PAS stained section) showing a positive staining colony

On macroscopic evaluation, the leg measured 30x11cm, the foot measured 27x10cm. The foot showed an indurated skin with some areas of hard abscess without any disharges. The initial sampling from these lesions showed a non specific inflammation without any tumoral lesion. Then, the remaining bone was submitted to decalcification by nitric acid. Some weeks later, after the process of decalcification, the macroscopic evaluation found a deep soft tissue and bone destruction consisted of round cavitis filled of yellowish crumbly material (Fig. 2). The histological examination on hematoxylin-eosine-safran (HES) stained sections revealed several multilobulated colonies surrounded by granulomatous inflammation composed of plasma cells, epithelioid cells, macrophages and some multinucleated cells. The colonies had deeply basophilic outer layers with branching filaments (Figs. 3 and 4); some colonies were fractured and had a pale center. They stained positive for PAS (Periodic Acid-Schiff) (Fig. 5). These histological aspects were strongly consistent with eumycotic mycetoma. The post-operative course was uneventful and the patient was discharged from the hospital. Two months after surgery, the patients had no signs of the disease and orthopedic prosthesis was prescribed for him.

## Discussion

Mycetoma is one of the neglected infectious diseases that is endemic in tropical and subtropical areas of the world [1, 5, 6]. The disease affects typically poor people living in rural areas and usually working in farms. Mycetoma affects all age groups, but it is more common in 20 – 40 year old men; this epidemiological feature suggests that young men are more exposed to the disease because they are supposed to be the more productive age group in developping countries [1, 3, 4]. The low prevalence of the disease in women could be due to hormonal factors as in rural areas women take part in agricultural and other activities that expose to mycetoma [1]. But, in countries out of the ‘’mycetoma belt”, in temperate climate regions, cases of mycetoma have been reported from immigrants [7–10]. Also, cases from autochtonous patients without history of travelling to endemic regions, have been reported [11]. Health practitionners in these areas are not familiar to the disease, thus cases were usually misdiagnosed and mismanaged leading to serious consequences for patients. In fact, our case, is from Morocco, a country that is out of the ‘’mycetoma belt”, where no more than 100 cases have been reported.

Several causative micro-organisms (more than 56), either fungi or bacteria, are known to date to be linked to mycetoma [1, 4, 6]. They are found in the environnment in plants thorns or in the soil. People become infected by the disease after injury by plants thorns or when walking bearfoot [4, 6]. The prevalence of causative agents vary in the world. In Sudan, the main causative agents are fungi, while in latin America, in countries like Mexico, bacterial agents are predominant. One recent review and meta-analysis, found that the species like *Actinomadura madurae, Streptomyces somaliensis, Actinomadura pelletieri, Nocardia brasiliensis* and *Nocardia asteroides* were considered to be common causative agents of actinomycetoma, while *Madurella mycetomatis* was the main causative agent of eumycetoma [1].

The clinical presentation of mycetoma is similar whether the causative agent is a fungi or a bacteria, however actinomycetoma has more agressive course and invades deeper structures earlier than eumycetoma [1]. Typically, patients present with a classical triad consisted of a painless firm subcutaneous mass, multiple sinus formation, and a purulent or seropurulent discharge containing grains. The disease pursues a long course, because of the indolor feature or the lack of appropriate health information about the disease, hence it occurs in poorly educated patients. Another factor that explains the long evolution of the disease, is the misdiagnosis especially in non endemic regions, as illustrated by our case that has disease for more than 20 years, repeatdly misdiagnosed, leading to leg amputation. Similarly, mycetoma cases have been reported in Europe, with long course and subsequent amputation [11].

The more challenging issue with mycetoma is the diagnosis in early stage of the disease before complications that could lead to aggressive therapeutic option such as amputation. The issue becomes even more challenging when it comes to identification of the causative agent. In fact, the treatment depends on the type of the causative microorganism and on the severity and extension of the disease. The imaging technics such as X-rays, ultrasonography, computed tomography (CT Scan) and magnetic resonance imaging (MRI) allow easily to assess the extension of mycetoma especially invasion of deeper structures like muscles or bones [13, 14, 16].

To identify the causative agents, culture methods are considered the gold standard as they allow the identification of the wide species linked to mycetoma [1, 13, 16]. However cultures methods are time consuming, certain species are difficult to identify, and contaminations are common. [1, 13, 14] The culture failed to identify the causative agent in our case, it only has identified *stapyloccocus aureus species.* Similarly, skin tests or serology could be used to identify the causative agents at species level, but these technics are not fully reliable [1, 13, 16]. Currently, molecular technics are the only reliable diagnostic tool to identify the exact species of the causative organisms. The main drawback of molecular technics is their high cost for developing countries where mycetoma is mostly endemic [1, 13]. Histopathology is another diagnostic tool that can aid to identify the causative agent. The main merits of pathology is to differentiate eumycetoma from actinomycetoma, identification at species level is not reliable, almost impossible [13–17]. Grains of the causative agent can be obtained by cotton swab from sinuses, by fine needle aspiration or by biopsy [1, 6, 16, 17]. Superficial grains from sinuses are often non viable and contaminated with other organisms, ideally deep-seated grains provide more diagnostic informations. The macroscopic examination of grains do not provide any specific diagnostic orientation. Eumycetoma could have black, white or yellow grains, whereas actinomycotic grains could have yellow, white, red or pink color. However, in a long standing disease, fibrotic lesions can be so extensive that discharge from sinuses becomes scarce or completely inapparent [14]. Biopsy from these lesions are always non conclusive or misleading, showing non specific inflammation or mimic certain malignancies, as commonly reported in the literature. In fact, the important population of reactive fibroblasts and histiocyts, along with fibrotic and haemorrhagic changes, may lead some pathologists to think about Kaposi sarcoma. The long course of the disease and its extension to adjacent structures may also play a role in the misdiagnosis of malignancies. Recently, in Morocco, another case has been reported where the patient had been misdiagnosed as Kaposi’s sarcoma, and given chemotherapy before the correct diagnosis of mycetoma [18]. Typically, our case illustrated also the diagnostic challenge posed by mycetoma especially in non endemic areas. The patient had several biopsies that showed non specific inflammation, the latest has concluded to Kaposi’s sarcoma invading bone structures, justifying amputation. The histopathological diagnostic approch uses hematein-eosin-safran (HES) stain combined with other special stain such as PAS, Gram stain, Ziehl Nielson stain (ZN), Grocott satin,…etc. [1, 13–17]. With HES stain, the grains represent colonies of the causative agent, surrounded by granulomatous inflammation composed of plasma cells, polymorphonuclear cells, macrophages and giant cells. Colonies from actinomycetoma have different size, often round or multilobulated, with deeply stained basophilic outer border and slightly paler center [14, 15, 18]. Sometimes, an eosinophilic hyaline-like material surrounds the colonies, this aspect is referred to as Splendore-Hoeppli Phenomenon [14]. The colonies may also show fractured aspect [1, 14, 15]. The filaments are thin, their thickness is no more than 1 μm [13–17]. Typically, actinomycotic colonies are Gram positive and negative for PAS stain [13, 14]. *Nocardia* species stain positively to ZN [1, 14]. Histologically, our case stained postive to PAS, the fact that allowed us to rule out actinomycetoma although colonies were multilobulated, fractured and had basophilic outer layers with pale centers, at HES stain. Colonies from eumycetoma show several histological aspects that can have overlapping appearence with actinomycetoma colonies, but their filaments are thicker, 2-6 μm [13, 14]. They stain positive for PAS, negative for Gram stain or ZN stain. In fact, as cultures are time-consuming, and sometimes negative, pathology provides useful aid to discriminate between actinomycotic and eumycotic causative agents, by using HES stain combined with other special stains [13–17]. Pathology also rule out any malignancy or specific granulomatous inflammations such us tuberculosis. Thus, treatment can be adjusted.

The treatment of mycetoma depends on the causative agent, either fungal or bacterial, hence the necessary determination of the type of causative organism. Both eumycetoma and actinomycetoma are treated with antifungal or antibacterial drugs, sometimes combined with surgery. The treatment of eumycetoma uses antifungal drugs belonging to the azole class, such as ketoconazole, itranonazole, terbinafine or voriconazole. But since 2013, there was some restrictions of the use of ketoconazole by the US Food and Drug Administration, followed latter by the European Medicines Agency, because of several side effects [1]. These drugs are used for months, and associated with surgical debridement. Recurrences are frequent, and compliance to treatment seems difficult [1, 4]. Actinomycetoma is treated by a combination of trimethoprim and sulfamethoxazole with aminosids (amikacin or netilmicine), for weeks. The prognosis of actinomycetoma seems to be better compared to eumycetoma [1, 6, 18]. Table 1 summarizes some different charactristics of eumycetoma and actinomycetoma.

**Table 1:** Differential characteristics between eumycetoma and actinomycetoma

	Eumycetoma	Actinomycetoma
Epidemiology	Africa, India	Latin America
Clinical course	Less aggressive	More aggressive
Grain	Black, yellow, white	Yellow, white, red, pink
PAS	Positive	Negative
Gram	Negative	Postive/Negative
Ziehl Nielson	Negative	Positive/Negative
Filaments	2-5 μm	<1 μm
Treatment	Antifungal (azole) + surgery	Antibacterials

Despite the long-term treatment and recurrences observed in medical treatment, aggressive surgery is not the first line of treatment [1, 6, 19]. Amputations are generally due to misdiagnosis or a long-standing disease that spreads to deeper structures of the body [5, 7, 11, 18]. In fact, our patient should have been treated medically, rather than surgically, but the misdiagnosis due to the fact that clinicians were not familiar to mycetoma here in Morocco, as it is not an endemic area of mycetoma. Misdiagnosis and mismanagement are common in non endemic regions.

## Conclusion

We have reported a case of mycetoma in a non endemic area, that evolved for more than 2 decades, misdiagnosed as a cancer and leading to an unnecessary and aggressive surgery. This case illustrated well the diagnostic challenge of mycetoma in certain areas of the world where the disease is not endemic;  health practitioners should be aware of that in order to provide early diagnosis and appropriate treatment.

## Abbreviations

HES: hematoxyline-eosine-safran
PAS: periodic acid-Schiff
WHO: World Health Organisation
ZN: Ziehl Nielson stain
